# Equity Effects of Dietary Nudging Field Experiments: Systematic Review

**DOI:** 10.3389/fpubh.2021.668998

**Published:** 2021-07-23

**Authors:** Benjamin Schüz, Hannah Meyerhof, Lisa Karla Hilz, Jutta Mata

**Affiliations:** ^1^Faculty 11, Institute of Public Health and Nursing Research, University of Bremen, Bremen, Germany; ^2^Department of Psychology, University of Mannheim, Mannheim, Germany

**Keywords:** equity effects, nudge, field experiment, dietary behaviour, nutrition behaviour, harvest plot, inequalities

## Abstract

**Background:** Dietary behaviours are among the key modifiable risk factors for non-communicable diseases. Importantly, dietary behaviours vary substantially between groups and individuals with different socioeconomic positions, with more disadvantaged groups and individuals being exposed to more dietary risk factors. The goal of this review is to summarise the existing research on equity effects of dietary nudging interventions.

**Methods:** Systematic review of nudging interventions conducted in a field setting that report an observable indicator of dietary behaviour, include a control group, and report effect sizes stratified by indicators of socioeconomic status as outlined in the PROGRESS-Plus framework. Two databases (scopus, Pubmed) were searched (last search June 2021), and 18 articles with 19 studies (k = 46 equity comparisons) were included. Risk of bias was assessed using the ROBINS-I tool. Due to heterogeneity in equity dimensions and study outcomes, a harvest plot was used to summarise data.

**Results:** The majority of equity comparisons (38 out of 46) were available for cognitive nudges. Most of these (22 out of 38 comparisons) found that cognitive nudges worked equally well in more and less disadvantaged populations; however, in 12 out of the 38 comparisons, they favoured those who were less disadvantaged. Two out of four comparisons on behavioural nudges favoured more disadvantaged persons.

**Conclusions:** The differential effects of dietary nudging interventions in this review can contribute to increases in health inequalities. At the same time, a substantial number of interventions showed no equity effects. Importantly, this review suggests that more research on nudging interventions and health equity is needed. Future interventions should report effect sizes stratified by indicators of social inequality.

**Systematic Review Registration:** PROSPERO (CRD42019137469)

## Introduction

Dietary behaviours remain among the key modifiable risk factors for non-communicable diseases such as cardiovascular disease, diabetes mellitus, or chronic obstructive pulmonary disease, thus contributing substantially to the global burden of disease (1). For example, non-communicable diseases account for between 33% (in low-income countries) to 88% (in high-income countries) of deaths (2).

However, dietary behaviours vary substantially between individuals and groups differing in socioeconomic position, with more disadvantaged groups and individuals being exposed to more dietary risk factors. For example, a systematic review (3) found that socioeconomically disadvantaged individuals and groups consume significantly less fibre, fruits, and vegetables, and that these differences were consistent by gender and region. Similar patterns were found for sugar intake, with higher total sugar intake in more disadvantaged groups [e.g., (4)]. At the same time, at least in the UK, women, older adults, and members of ethnic minorities are more likely to adhere to dietary recommendations (5).

These socioeconomic differences in dietary behaviours account for about 21% of the differences in all-cause mortality according to education (6).

A number of factors are discussed as being relevant in socioeconomic and sociodemographic differences in dietary behaviours, among them the relatively higher costs of foods that are nutrient-rich and low in energy density (7), differences in health literacy (8), as well as differences in the exposure to fast food outlets, food advertisements, and food availability in the proximal food environment such as neighbourhoods (9). In terms of psychosocial determinants of individual dietary behaviour, a systematic review suggests that study-level socioeconomic status did not moderate the effects of intentions and perceived behavioural control on dietary behaviours (10), but it is possible that socioeconomic differences in these determinants translate into differences in dietary behaviours (11).

An influential review (12) identified 6 components of effective dietary interventions on individual or population level:—price, place, product, prescriptive, promotion, and person. Here, “price” describes interventions that aim at changing the price of foodstuffs in order to increase or decrease consumption, “place” describes interventions that prescribe point-of-sales for food outlets, “product” describes interventions that aim at changing the health-relevant ingredients of foodstuffs, “prescriptive” those interventions that restrict advertising or marketing, “promotion” those that use mass media campaigns to encourage or discourage specific dietary behaviours, and “person” those that aim at person-based information and education.

More importantly, this review (12) also identified socioeconomic and sociodemographic differences in the effects of interventions in that interventions that targeted price components were more likely to improve dietary behaviours in more disadvantaged populations, whereas interventions that targeted information and person-level education were more likely to improve dietary behaviours in less disadvantaged populations.

Such differential effects (so-called “equity effects”) of public health interventions according to socioeconomic position pose a considerable challenge to population health interventions promotion and disease prevention, as they indicate that interventions to improve health behaviours could inadvertently increase health inequalities (12–15). A logic model framework (13) suggests that equity effects of public health interventions could be due to differential recruitment into interventions, differential engagement with intervention material, differential retention, or differential effects of intervention components. For example, interventions that require cognitive effort in acquiring, understanding, and acting on nutrition-related information are more likely to be effective in less disadvantaged populations, because the intervention components require considerable individual agency, which in turn might be less available to more disadvantaged individuals or groups (16).

One particular behavioural strategy to change dietary behaviours that has been discussed in terms of low requirements of individual agency (17) is nudging, that is, a modification in the so-called “choice architecture” that changes individual dietary behaviour without nominally restricting alternative behavioural choices (18). This “choice architecture” involves micro-environments (19), that is, those small-scale localised social and physical environments (e.g., restaurants, workplaces, shops) that provide cues and stimuli for dietary choices. The (more or less implicit and often not explicitly tested) assumption about the effects of nudging include that nudges operate via a non-conscious process of behavioural regulation based on heuristic processing instead on conscious, deliberate behavioural decisions, and that processing nudges thus requires less cognitive resources than making conscious decisions (20). This would mean that, for example, making dietary choices in a take-away environment in which “healthier” meals are promoted via heuristics such as better accessibility or social proof (i.e., stressing that the “healthy” choice was the most popular choice) should lead to a higher frequency of the nudged choices (21).

However, to date, the majority of studies examining the effects of nudging on dietary behaviours have been conducted in controlled or laboratory settings. In order to establish the scaleability of nudging to population level for population health effects, it is crucial to establish the effects of nudging interventions in real-world settings and, more relevant for this study, to identify potential equity effects in real-world settings in order to gauge potential benefits or intervention-generated inequities for disadvantaged populations (16). The evaluation of nudging effects on dietary outcomes in real-world settings is further more likely to include participants from more diverse backgrounds than those that are most often recruited for laboratory-based research.

A recent meta-analysis of field experiments on nudging dietary behaviours (18) found consistent small to moderate effect sizes for nudges in real-world settings (as opposed to previous reviews that also included laboratory studies) for cognitively oriented (i.e., nudges that provide easily accessible information about the food, for example, via traffic-light symbols; *d* =.12), affectively-oriented nudges (e.g., changing the hedonic value of specific foods; *d* = 0.24), and behaviourally-oriented nudges (e.g., changing the accessibility or convenience of food choices; *d* = 0.39). Alternative typologies and classifications of nudges exist (19, 20, 22–25), but these are often based on the intervention format and technique [e.g., (24)] or the intended function of the nudging intervention [e.g., (19)], whereas the Cadario and Chandon meta-analysis (18) employs a tripartite model of behavioural regulation which allows integrating behavioural theory.

As nudging is increasingly being discussed as policy instrument to modify individual health-related behaviours (23, 26), it is crucial to evaluate if the effects of nudging on behaviour are equal across socioeconomic and sociodemographic groups, or whether nudging interventions show equity effects (12–15) similar to conventional dietary interventions (12).

In this systematic review, we aim at providing such equity analyses of existing real-world interventions that employ nudging strategies to change dietary behaviours. We utilise the established classification of dietary nudges into cognitively-oriented, affectively-oriented, and behaviourally-oriented (18) and examine whether the effects of these types of nudges differ by the facets of social inequality. Here, we use the PROGRESS-PLUS framework (27) to identify relevant dimensions of social inequality. Proposed by the Campbell and Cochrane Equity Methods Group, the acronym PROGRESS represents eight dimensions across which inequalities may exist: Place of residence, Race/ethnicity/culture/language, Occupation, Gender/sex, Religion, Education, Socioeconomic status and Social capital. “Plus” considers other characteristics of populations which may be associated with social disadvantage (e.g., age, disability or sexual orientation). This allows a comparable operationalisation and identification of potential working mechanisms of inequality, thus enabling an equity-focused review (15, 28) of the effects of nudging interventions on dietary behaviours in real-world settings.

## Method

This review had the primary aim to examine equity effects in field experiments of dietary nudging interventions. We defined a field experiment as any study comparing one or more groups receiving any nudging intervention that was consistent with the definition of a nudge in (18) to any type of control group in a natural setting (29). This excluded studies in simulation settings such as fake food buffets (30) or laboratory restaurants (31). The review was conducted in accordance with the PRISMA statement (28, 32). The review was registered with PROSPERO (CRD42019137469).

### Literature Search

Searches were conducted in four electronic databases (Medline, Google Scholar, Scopus, PsycINFO) with a final search in June 2021 in accordance with the search strategy piloted and published by Cadario and Chandon (18), combining search terms for dietary behaviours, nudging, field studies, and behavioural outcomes. Searches were limited to manuscripts available in English or German, and to those published after 2018 (so as not to duplicate the search results from Cadario and Chandon). The search strategy for scopus is available in online Supplementary Material 1.

### Inclusion Criteria

To be included in the review and equity analysis, studies had to (a) employ a nudging intervention consistent with the Cadario and Chandon definition (18); (b) be conducted in a field setting in which adult participants conducted everyday interactions unaffected by the intervention (i.e., not be conducted in settings specifically designed for study purposes); (c) report an observable indicator of food selection or consumption; (d) compare the nudging intervention against a control group; and (e) report effect sizes stratified by any PROGRESS-Plus dimension, either on individual or site level.

### Screening and Selection

In a first step, duplicates resulting from the literature search in multiple databases were removed (*n* = 12). Next, titles and abstracts of *n* = 1,333 articles were screened by one reviewer (HM), and a 10% random sample was checked for consistency by a second reviewer (BS) with an agreement of 89.55%. During this step, inclusion criteria were applied and any articles that could clearly be identified as not fulfilling inclusion criteria was discarded (*n* = 1,276).

Following this, the full texts of the remaining 57 articles and the 90 articles included in Cadario and Chandon were retrieved and screened by two reviewers (HM and CK) for reporting of effect sizes stratified by any PROGRESS-Plus indicator (27). The final sample of *n* = 19 studies in 18 articles were then subject to data extraction (see below) by two independent reviewers (HM and BS).

### Supplementary Searches for Associated Publications and Effect Sizes

For each study that met inclusion criteria, steps were initiated to retrieve additional publications from the data set to examine potential equity analyses. In particular, publications were searched using trial registration numbers where applicable, and forward citation tracking in Google Scholar was applied to identify potential follow-up publications. Authors of studies that reported data necessary for equity analyses but did not report equity analyses were contacted via e-mail, and one additional study (33) was included after authors provided results from equity analyses.

### Data Extraction

For each study that met inclusion criteria, effect sizes, study characteristics, and basic study information were extracted (summary in Table 1). References for studies were managed using ThomsonReuter EndNote X8. Study data were extracted in parallel by HM and BS. The data extracted included name of the authors, publication year, journal of main study publication, study population and sample size, setting, nudging type, type of dependent variable, level of dependent variable (individual vs. location), PROGRESS-Plus dimensions reported, level of PROGRESS-Plus dimensions (individual vs. location), type of equity analysis [following Sun et al. (50), these were defined as either interaction or subgroup analysis], overall effect size, and effect sizes by PROGRESS-Plus dimension.

**Table 1:** Studies included in review.

**References**	**Outcome for equity analyses**	**Nudge type and content**	**Sample size**	**PROGRESS-Plus dimensions**	**Results (Equity effects only)**	**Statistical test for equity effects**
1. Auchincloss et al. (34)	Self-reported food ordering behaviour	Cognitively oriented nudge: Descriptive nutritional labelling	648	Sex Age Race/Ethnicity Socioeconomic Status (Income) Educational Attainment	- No differences between men and women in self-reported ordering - Older adults more likely to report changes in ordering - African Americans less likely to report changes in ordering behaviour - Participants with lower income less likely to report changes in ordering behaviour - Participants with lower educational attainment less likely to report changes in ordering behaviour	Chi squared tests for frequency table
2. Bauer et al. (35)	Purchases of healthy food option	Behaviourally oriented nudge: Convenience	2,226 cafeteria patrons	Occupation	- Regular employees chose healthy food option significantly more often after intervention. - No significant pre-post differences in trainees, interns and guests - No significant between-group differences	None
3. Bauer et al. (35)	Purchases of healthy food option	Cognitively oriented nudge: Visibility enhancement		Occupation	- Interns chose healthy option significantly less often after intervention compared to other occupation groups	None
4. Bollinger et al. (36)	Energy content of customer purchases	Cognitively oriented nudge: Descriptive nutritional labelling	216 café locations	Socioeconomic status (income) Educational Attainment Age Sex	- Lower log calories per transaction after posting in ZIP areas with higher than median income - Lower log calories per transaction after posting in ZIP areas with higher proportion of inhabitants with college degrees - No difference in log calories per transaction after posting in ZIP areas differing in proportion of younger adults (20–45) - No difference in log calories per transaction after posting in ZIP areas with higher proportion of females	Interaction term in multiple regression
5. Cawley et al. (37)	Energy content of customer purchases	Cognitively oriented nudge: Descriptive nutritional labelling	5,551 restaurant patrons	Sex Race/Ethnicity Occupation	- No significant differences in total calories between genders - No significant differences in total calories between white and non-white patrons - No significant differences in total calories between college students and other occupations	Interaction term in regression
6. Crockett et al. (38)	Energy consumption	Cognitively oriented nudge: Evaluative nutritional labelling	287 participants	Place of residence (area level of deprivation)	- No significant two-way interactions area level of deprivation and nudge type	Interaction term in multiple regression
7. Elbel et al. (39)	Energy content of customer purchases	Cognitively oriented nudge: Descriptive nutritional labelling	1,156 participants	Place of residence Sex Age Race (Ethnicity; African American/Latino)	- No significant interactions of pre/post labelling with place of residence, sex, age, or ethnicity	Test unclear
8. Elbel et al. (40)	Energy content of customer purchases	Cognitively oriented nudge: Descriptive nutritional labelling	2,083 purchases	Age Sex Ethnicity Education	- No significant differences between experimental (Philadelphia) and control (Baltimore) city in energy of fast food purchases - No significant effects in any subgroup by PROGRESS-Plus	Subgroup analyses without *post-hoc* comparisons
9. Freedman (41)	Self-reported buying behaviour	Affectively oriented: Healthy Eating Call (e.g. “Portion size matters”)	1,675 students	Sex	- A significantly higher proportion of women compared to men reported being influenced by healthy eating calls	Chi-squared test on frequency table
10. Krieger et al. (42)	Energy content of customer purchases	Cognitively oriented nudge: Descriptive nutritional labelling	7,325 customers	Place of residence Sex Age Race/Ethnicity	Both in food chains and coffee chains: - Bigger decrease in caloric content after nudge in females compared to males, - Bigger decrease in white participants compared to non-white participants - Bigger decrease in participants living in more affluent areas compared to less affluent areas. - No age differences.	Subgroup analyses with *post-hoc* comparisons
11. Levy et al. (43)	Purchases of labelled food	Cognitively oriented nudge: Evaluative nutritional labelling and visibility enhancements	4,642 employees (53,371 transactions).	Race/ethnicity Occupation	- Significant overall decreases of purchases of items with red labels after evaluative labelling - No interactions of evaluative labelling with race / ethnicity - Significantly lower purchases of beverages in professional staff compared to managerial staff after evaluative labelling - Significantly lower purchases of beverages in managerial staff compared to all other occupational groups after visibility enhancements	Interaction term in multiple regression
12. Mistura et al. (44)	Vegetable purchases	Cognitively oriented nudge Visibility enhancement	24,410 purchases	Sex	- No significant effects of interventions in male or female students	Subgroup analysis without *post-hoc* adjustment
13. Oliveira et al. (45)	Food purchases	Cognitively oriented nudge: Evaluative nutritional labelling and descriptive nutritional labelling	233 students	Sex	- Significant increases in healthy food orders after cognitively oriented nudge in females only	Subgroup analysis without *post-hoc* adjustment
14. Payne et al. (5)	Food purchases (healthy micro-packs)	Behaviourally oriented nudge: Convenience enhancement	Checkout data from 3 stores	Socioeconomic status (eligibility for Supplemental Nutrition Assistance Program; SNAP)	- Bigger increase in healthy micropack sales in SNAP transactions compared to non-SNAP transactions	None
15. Polacsek et al. (33)	Food purchases (weekly spending on fruit and vegetable)	Behaviourally oriented nudge: Convenience enhancement	401 supermarket customers	Socioeconomic status (eligibility for Supplemental Nutrition Assistance Program; SNAP)	- SNAP participants were less likely to redeem coupons - SNAP participants who redeemed coupons had higher income than SNAP participants who did not redeem coupons - SNAP participants who redeemed coupons spent more on healthy food items than non-SNAP participants redeeming coupons	Subgroup analysis with *post-hoc* adjustment
16. Salmivaara et al. (46)	Purchases of sustainable food option	Affectively oriented nudge: Healthy eating call	1,289 patrons	Age Sex Education	- Stronger effects in older educated females and younger males	Fuzzy-set qualitative comparative analyses (fQCA)
17. Thorndike et al. (47)	Purchases of labelled beverages	Cognitively oriented nudge: Evaluative nutritional labelling	2,285 participants	Race/Ethnicity Occupation	- Significant overall decreases in purchases of unhealthy (red labelled) beverages - Smaller decreases in purchases of unhealthy (red labelled) beverages in Black and Latino compared to Asian and White employees - Smaller decreases in purchases of unhealthy (red labelled) beverages in Service Workers compared to Management / Clinician - Bigger increase in purchases of healthy (green labelled) beverages in Asian, Latino and Black compared to White employees - Bigger increase in purchases of healthy (green labelled) beverages in technicians compared to other professions	None
18. Vanderlee et al. (48)	Energy consumption	Cognitively oriented nudge: Descriptive nutritional labelling and evaluative nutritional labelling	1,003 participants	Race/ethnicity Sex Educational attainment Socioeconomic status (income) Age	- Significant overall decreases in energy consumption after labelling - No interaction effects between labelling and race/ethnicity, sex, educational attainment, income, and age.	Interaction term in multiple regression
19. Vermote et al. (49)	Fruit purchases	Cognitively oriented nudge: Evaluative nutritional labelling	33,386 sales in university restaurant	Sex Educational attainment	- No differences in effects between male and female students as well as staff - More fruit purchases over longer time periods in staff with higher educational attainment	None

In 17 out of 19 studies, there were no discrepancies (89.47% congruence between raters), and all remaining discrepancies in data extraction were resolved through discussion.

### Quality Appraisal

Study quality was appraised using the Cochrane Collaboration Risk of Bias in Non-randomised Studies tool [ROBINS-I; (51)]. This tool specifies seven domains of risk of bias, specifically bias arising from confounders, bias due to selection of participants, bias in the classification of interventions, bias due to deviations from intended interventions, bias due to missing data, bias in measurement of outcomes, and in the selection of the reported results. Applying the ROBINS-I tool resulted in an overall risk of bias score per study and identifies specific areas of concern per study. Studies were assessed by two independent reviewers, and scoring discrepancies were resolved through discussion between the reviewers.

### Analysis

The protocol for the review suggested either qualitative narrative review or quantitative meta-analysis for the analysis of identified studies. As the number of studies reporting equity effects of nudging interventions was small (*n* = 19) and heterogeneous both with regards to PROGRESS-Plus dimensions and outcome measures, both narrative and graphical [harvest plots; (52)] methods were used to synthesise the results of the studies in the review.

## Results

**Figure 1:**
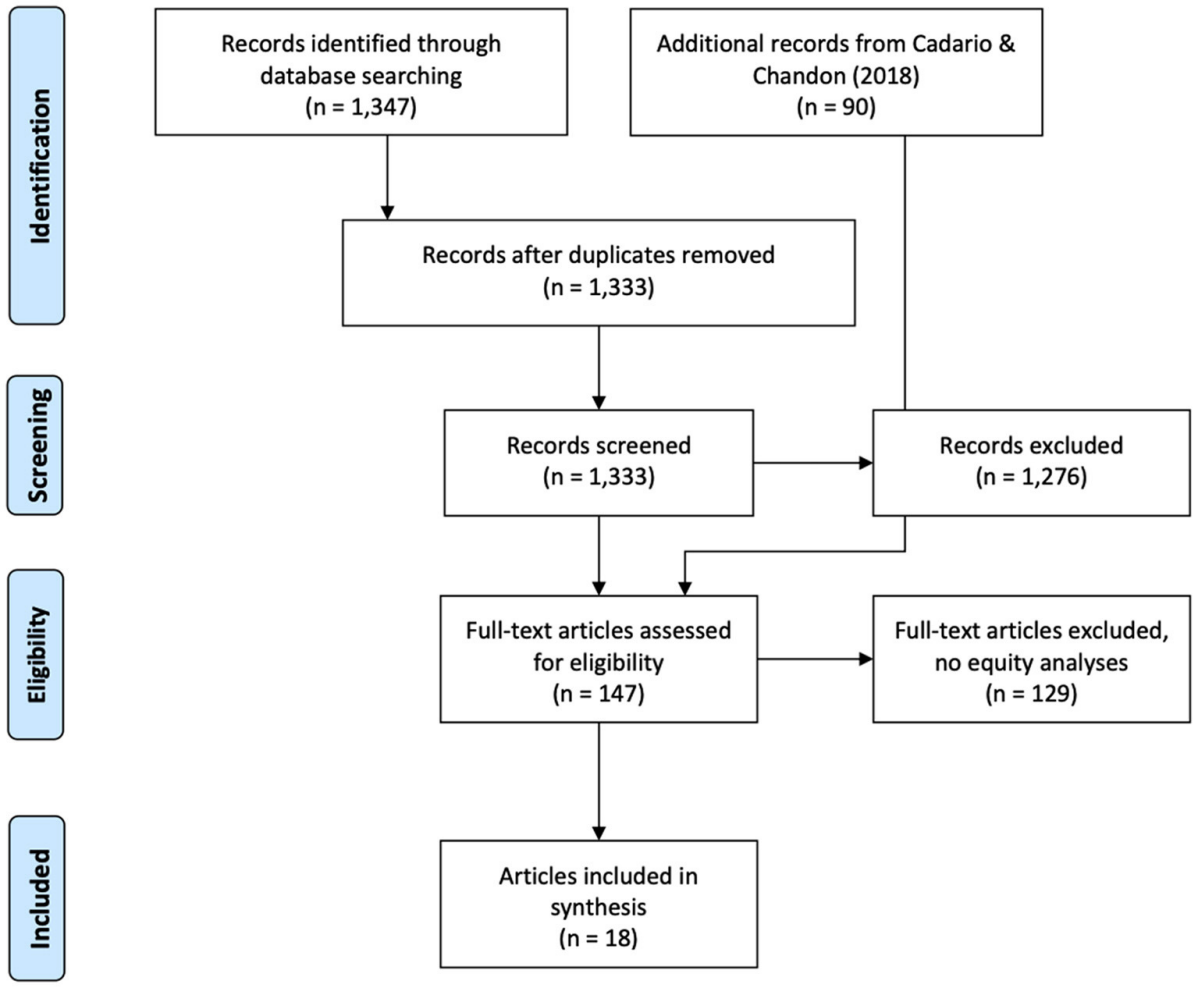
PRISMA flow diagram.

The results of the screening and identification process can be found in Figure 1. Using the search terms outlined above, and limiting the search to studies published in or after 2018, 1,333 records were identified. Of these, 1,276 were excluded on the basis of not meeting inclusion criteria. This means that, together with the 90 articles examined in the Cadario and Chandon (18) review, 147 articles were identified that could potentially report equity analyses of field experiments of dietary nudging interventions. We subsequently obtained full-texts of 147 articles and assessed these for eligibility for the review. Of these, 129 (87.76%) had not performed equity analyses, but 18 (12.24%) reported results stratified by any PROGRESS-Plus indicator. We subsequently contacted authors of these studies enquiring if equity analyses had been performed or would be possible. Of those authors who responded to our request, 12 indicated that equity analyses would not be feasible, and authors of one study (33) provided additional equity-related results, which were subsequently included in the review. The total sample of studies in the review accordingly consisted of *n* = 18 publications with 19 studies (33–49, 53) providing 32 comparisons of nudging effects across PROGRESS-Plus indicators. A summary is provided in Table 1.

### Quality Appraisal

The majority of studies (17 out of 19) could be classified as “low” risk of bias according to the ROBINS-I tool (51). However, most studies (16 of 19) used an objective measure of dietary behaviours by either examining purchase records, purchase receipts, or by behavioural observation. Only three studies (34, 41, 46) relied on self-reports of behaviour. Eight out of the 19 studies did not provide formal tests for equity effects, six further studies provided subgroup analyses, and only five out of 19 studies (36–38, 43, 48) provided a formal test (interaction term in regression analyses). An overview of the quality appraisal and risk of bias can be found in online Supplementary Material 2.

### Types of Nudges

The majority of studies in the review (14 of 19) employed cognitively oriented nudges, with 6 studies (34, 36, 37, 39, 40, 42) providing descriptive nutritional labelling only (e.g., mandated calorie labelling on the menu board and printed menus in fast food chains; 40), three studies (38, 43, 47) providing evaluative nutritional labelling only (e.g., traffic-light labelling of cafeteria items based on main ingredients, saturated fat and caloric content; 46), 3 studies (43, 45, 48) providing a combination of descriptive and evaluative labelling (e.g., a combination of nutrition labelling and health logos for healthier options in hospital cafeterias; 47), and one study providing visibility enhancements (44). Two studies (39, 46) used an affectively oriented nudge (healty eating calls such as “Portion size matters”), and four studies [(33, 45, 54); study 2 and 3] used behaviourally oriented nudges, such as convenience enhancement.

### PROGRESS-Plus Dimensions

**Figure 2:**
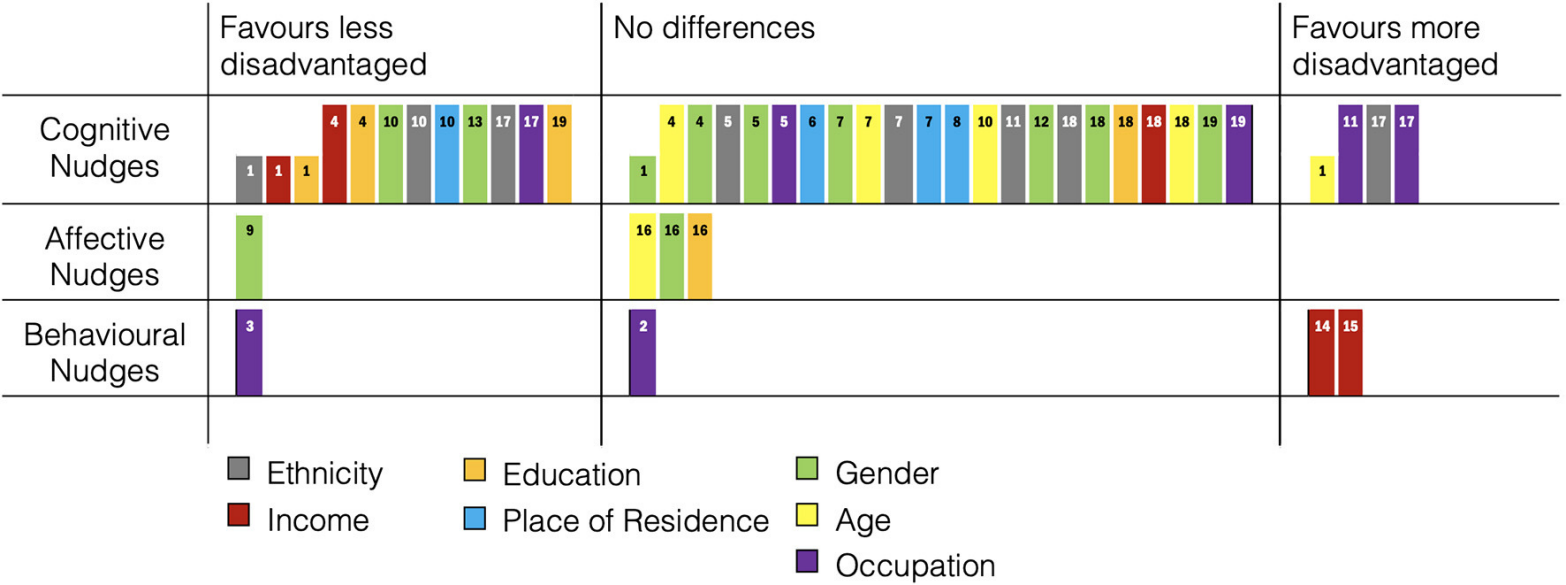
Harvest Plot: Equity comparisons of dietary nudges. Column height represents dietary assessment (low = self-report, high = objective). Numbers in columns refer to study number in Table 1.

Studies examined gender (11 out of 46 comparisons; 23.91%), race/ethnicity (8/46; 17.39%), occupation (7/46; 15.21%) age (6/46; 13.04%), income (5/46; 10.87%), education (5/46; 10.87%), and place of residence (4/46; 8.26%) from the PROGRESS-Plus dimensions. There were no systematic differences of equity effects (favouring less and more disadvantaged) by PROGRESS-Plus dimensions (Figure 2).

### Overall Effects of Nudges

The majority of studies (14 out of 19) reported at least one significant overall effect of nudging on dietary behaviour, with five studies [38, 40, 50; study 2, 51, 52] reporting no significant differences between study sites or experimental conditions. In all studies with significant effects, nudging was associated with increases in the indicators of healthy dietary behaviours.

### Equity Effects

A summary of the equity comparisons in the studies of this review is provided in Figure 2.

### Studies With Equal Effects Across PROGRESS-Plus Dimensions

Out of 46 equity comparisons, 26 (56.52%) showed no differences between PROGRESS-Plus dimensions (34, 36–40, 42–44, 48, 49). Most of these studies examined cognitively oriented nudges, while (46) examined an affectively oriented nudge, and [50; study 2] examined a behaviourally oriented nudge.

### Studies With Effects Favouring Less Disadvantaged Populations

Fourteen comparisons (30.43%) indicate more favourable effects of nudging in less disadvantaged participants (23, 34, 36, 41, 42, 45, 47, 49). These include one study with affectively oriented nudges (41), with the rest of the studies employing cognitively oriented nudges.

Studies with cognitively oriented nudges employed either descriptive or evaluative nutritional labelling, that is, additional information on the energy and nutrition content of the foodstuffs or beverages. The PROGRESS-Plus dimensions most affected by this are ethnicity, gender, education, and income. In particular, Auchincloss et al. (34) employed descriptive nutritional labelling and examined whether this affected self-reported food ordering behaviour. Older adults (indicator for more disadvantaged population group) reported more changes in ordering behaviour, whereas African American participants, those with lower educational attainment, and those with lower income were less likely to report changes in food ordering. Bollinger et al. (36) estimated the energy content of orders in chain coffee stores before and after descriptive nutritional labelling. They found that energy content decreased more strongly in ZIP areas with higher income and higher proportions of college graduates. No significant differences were found between ZIP areas differing in proportion of older adults and females. Krieger et al. (42) examined effects of menu labelling (cognitively oriented nudge) and found bigger decreases in the energy content of purchases in female participants compared to men, in white participants compared to black participants, and in participants living in more affluent areas, with no age differences. Oliveira et al. (45) found more healthy food choices in female customers following an evaluative nutritional label. Thorndike et al. (47) examined an evaluative nutritional label (red vs. green) and reported smaller decreases in red labelled (unhealthy) purchases in African American compared to other ethnicities, smaller decreases in lower-skilled as compared to other workers, but at the same time larger increases in the purchases of healthy (green labelled) foodstuffs in African American compared to other ethnicities.

Freedman (41) used healthy eating calls (an affectively oriented nudge) and reported a higher proportion of females as compared to males to report being influenced by this healthy eating call.

Bauer (35) in study three employed a behaviourally oriented nudge (convenience enhancement) and found mainly those in less precarious employment to choose healthy food options more often.

### Studies With Effects Favouring More Disadvantaged Populations

Six comparisons (13.04%) indicate more favourable effects of nudging in more disadvantaged participants. Two of these studies (34, 47) examined cognitively oriented nudges, and in both of these studies, the comparison favouring more disadvantaged participants was contrasted with findings that favoured less disadvantaged participants, see section above.

Two studies examining behaviourally oriented nudges reported more favourable outcomes in more disadvantaged participants (33, 53) characterised in both studies through lower socioeconomic position indicated through eligibility for the Supplemental Nutritional Assistance Program (SNAP) in the US. Payne et al. (53) found that placing micro packs of healthy nutritional items (fruits and vegetables) next to the checkout and indicating the eligibility of SNAP customers to purchase these led to larger increases in sales of these micro packs in SNAP-eligible customers compared to non-SNAP customers. Polacsek et al. (33) examined whether a convenience enhancement nudge (2 for 1 program for particularly healthy food stuffs) led to increased purchases of fruits and vegetables. They found that amongst those SNAP-eligible customers who participated in the program, a higher proportion of the weekly shopping budget was spent on fruits and vegetables. However, those SNAP customers that participated in the program had higher incomes than non-participating SNAP customers.

## Discussion

This systematic review examined equity effects of dietary nudging interventions in field settings, that is, whether the effects of nudging interventions on dietary behaviours varies between individuals or groups with different indicators of socioeconomic position. Nudging interventions were classified according to a previous systematic review (18) into cognitively oriented nudges, affectively oriented nudges, and behaviourally oriented nudges. Socioeconomic and sociodemographic indicators were based on the PROGRESS-Plus framework (27), which outlines dimensions of social stratification that are relevant for health inequalities.

Overall, compared to the almost 100 studies included in the previous review (18), only very few studies reported any analyses that were stratified according to any PROGRESS-Plus indicator, and only 13 studies could be included into the present equity-focused review.

### Equity Effects in Cognitively Oriented Nudges

The degree to which nudging interventions for dietary behaviours have different effects according to indicators of social inequality appears to differ by the type of nudging employed. Similar to a previous review (18) the current study is based on, the majority of nudges were classified as “cognitively oriented”, that is, these nudges provided some kind of information or other input that was designed to produce changes in cognitions relevant for changes in dietary behaviours. The majority of comparisons between individuals characterised by differences in PROGRESS-Plus indicators found that these interventions worked equally well in more and less disadvantaged populations (36, 38–40, 43, 48). However, a substantial proportion of these cognitively-oriented nudges also produced larger effects in terms of increases in healthier dietary behaviours in less disadvantaged individuals (34, 36, 42, 45, 47). Here, “less disadvantaged” meant groups characterised by ethnic majority (mostly white), female gender [as gender differences in nutrition imply less healthy dietary patterns and less adherence to dietary recommendations in males; (5)], higher educational attainment, and higher income. Such differential effects are a potential problem, as such equity effects might contribute to increases in health inequalities (12, 14, 15, 28): If a nudging intervention to change dietary behaviours is more effective in less disadvantaged individuals or population groups, this could, via the health effects of dietary behaviours, contribute to an increase in the difference in health outcomes between less and more disadvantaged individuals and groups—even if the uptake and retention of such interventions does not differ between groups (13).

In particular for cognitively oriented nudges, which are characterised for example, by nutritional information on the dietary items, traffic-light oriented evaluative information, or calorie postings, the effects on dietary changes are assumed to be mediated in changes in cognitions, which in turn are assumed to affect dietary choices—that is, dietary decisions (22). Whether and to which degree decisions to change health-related behaviours, however, is dependent on individual agency (16) and resources required to enact such decisions (37), both of which are unequally distributed along PROGRESS-Plus dimensions. This would imply that even though cognitively-oriented nudging interventions have demonstrated effects on changing dietary behaviours in field experiments (18), these effects are more likely to occur in individuals who are less disadvantaged. This finding is somewhat in line with previous studies on socioecononomic inequalities in the effects of dietary interventions (12) that found in particular low-agency interventions targeting the price of foodstuffs to reduce social inequalities in healthy eating outcomes.

However, the substantial number of intervention comparisons in our review that found no equity effects suggests that this is not a general pattern, but that further moderating variables on study and setting level might be responsible for facilitating or buffering equity effects.

### Equity Effects in Behaviourally Oriented Nudges

Two of the studies in this review (33, 53) employed behaviourally oriented nudges, in that both studies facilitated access to fruits and vegetables either through placement close to checkout counters and convenience enhancement (53), or through a 2-for-1 program (33). Both studies reported stronger effects in terms of purchases of healthier foodstuffs in more disadvantaged participants [in both cases participants eligible for the U.S. Supplementary Nutrition Assistance Program (55)]. This means that these interventions could have the potential, again via the health effects of healthier dietary items, to decrease health inequalities (15). However, whether such relatively isolated effects on purchases of fruits and vegetables really have beneficial effects on health inequalities in the long run cannot be ascertained with studies such as this. Further, the 2-for-1 program (33) found differences in participation according to PROGRESS-Plus factors in that more disadvantaged participants were less likely to participate in the program in the first place, thus potentially producing equity effects via differential participation (13). Without intention-to-treat analyses based on enrolment eligibility, such equity effects cannot be ruled out.

Nevertheless, these findings are in line with wider findings regarding equity effects of interventions that require higher levels of individual agency (16) as both behaviourally oriented nudge studies employed strategies requiring relatively little individual agency to change dietary behavioural outcomes.

### Affectively Oriented Nudges

Only one study (41) employed an affectively oriented nudge in Cadario and Chandon's (18) classification, and this study found female as opposed to male students to profit more from a healthy eating call. As this is the only study in our review that employed such a strategy, we refrain from interpreting this further.

### Dimensions of Inequality Implied in Equity Effects

All PROGRESS-Plus dimensions observed in the studies in the review were implied in equity effects. However, only occupation, income, ethnicity and age were implied in studies that reported effects favouring those more disadvantaged. Two studies (33, 53) reported effects of behavioural interventions favouring those with lower income, but at the same time, two studies (34, 36) found effects of cognitively oriented nudges favouring those less disadvantaged. Education, place of residence and gender were only implied in interventions showing effects that favoured those less disadvantaged. Overall, these differential effects are very heterogeneous, and more research systematically examining the PROGRESS-Plus dimensions of inequality that could affect equity effects of interventions are needed.

### Limitations

This review is subject to a range of potential limitations, arising both from the conceptualisation of the review and the studies in the review. First, the study selection for this review is an updated version of studies included in a previous review (18) and thus shares the limitations inherent in this particular review. However, as the focus of this study were nudging interventions for dietary behaviours in real-world settings, it seemed prudent to replicate the search strategy of this previous review.

Second, while the classification of nudges into cognitively oriented, affectively oriented, and behaviourally oriented has substantial face validity, it remains silent as to the underlying behaviour change mechanisms, in particular with regard to cognitive mechanisms. Alternative nudging classifications (19, 23–25) that focus on the delivery method rather than the content could potentially yield different findings. At the same time, the relatively small number of studies that provided any information on socioeconomic differences makes a finer-graded differentiation of nudging types even more problematic than in the current study.

Third, the studies examined are very heterogeneous in terms of study populations, target behaviours, settings, and study designs. Only two studies (38, 47) randomly assigned participants into nudging and control groups, whereas the majority of studies used either non-random control sites or controlled pre-post designs. While this is plausible given the scope of the review (real-world settings rather than laboratory settings), it is a major limitation for the interpretation of any nudging effect as causal.

Fourth, the relative paucity of evidence is a particular challenge for establishing equity effects (or the absence thereof), as only a fraction of the relevant population of study effects could be included in this review, because very few studies reported effects stratified by any PROGRESS-Plus indicator. This paucity of effect sizes available for review is however a general problem of equity-focused reviews (13, 28, 56), and only more inclusive reporting of effects in subpopulations stratified by PROGRESS-Plus indicators can solve this issue. There are calls for a more thorough reporting of effect sizes (28), but unless enforced by publications, collecting evidence for equity effects for behavioural interventions will remain piecemeal.

### Future Research

There are several avenues for future research based on the current review. First, the majority of studies reviewed did not report effect sizes stratified by any indicator of social inequality. This is a substantial problem for examining such equity effects, and future studies on nudging interventions [or, more broadly, on any behaviour change intervention; (56)] should routinely report such potential differences. With an increasing evidence base for equity effects of dietary nudging interventions, a more thorough and updated version of the current review could be conducted to allow for a more precise estimation of equity effects of nudging. Second, it needs to be examined whether classifying nudges according to different typologies [e.g., (19, 24, 54)] would yield similar findings. Further examination of nudging equity effects on different intervention levels [from targeting the individual to meso- and macro-level interventions; (57)] could further yield additional information on which nudging interventions are most likely to produce differential effects according to socioeconomic position. Further, a more thorough classification of the mediating processes underlying nudging effects would allow a finer-grained examination of the mechanisms underlying equity effects of nudging interventions.

### Implications and Conclusions

The main implication of the current review is that nudging interventions for dietary behaviours might be subject to similar differences in their effects as conventional interventions to change dietary behaviours (12), namely that particularly those nudges that rely on providing information are more likely to disproportionally benefit less disadvantaged participants, both individuals and groups. At the same time, low-agency (16) nudging interventions that focus on facilitating behaviour are more likely to benefit more disadvantaged populations (12). Practical implications include that both health promotion professionals and health policy makers might consider the potential of low-agency interventions, potentially those that combine behaviourally oriented nudges with price incentives to reduce socioeconomic and sociodemographic differences in nutritional behaviours.

However, and most importantly, more research on equity effects in health intervention research and its underlying mechanisms is dearly needed to eventually design effective public health interventions that can improve dietary behaviours for public health.

## Data Availability Statement

The original contributions presented in the study are included in the article/Supplementary Material, further inquiries can be directed to the corresponding author/s.

## Author Contributions

BS: conceived of the study, conceptualised study protocol, contributed to data collection, analysed data, and wrote first manuscript draft. HM: contributed to study protocol, organised data collection, analysed data, and edited manuscript. LH: contributed to study protocol, data interpretation, and edited manuscript. JM: contributed to study concept, contributed to study protocol, contributed to data interpretation, edited manuscript. All authors contributed to the article and approved the submitted version.

## Conflict of Interest

The authors declare that the research was conducted in the absence of any commercial or financial relationships that could be construed as a potential conflict of interest.

## Publisher's Note

All claims expressed in this article are solely those of the authors and do not necessarily represent those of their affiliated organizations, or those of the publisher, the editors and the reviewers. Any product that may be evaluated in this article, or claim that may be made by its manufacturer, is not guaranteed or endorsed by the publisher.

## Supplementary Material

Data Sheet 1 Search terms.

Data Sheet 2 Risk of bias (ROBINS-I).
