# Supplementary material for: Equity Effects of Dietary Nudging Field Experiments: Systematic Review
Source: Front Public Health. 2021 Jul 23;9:668998. doi: 10.3389/fpubh.2021.668998 (PMC8342848; doi:10.3389/fpubh.2021.668998)
Supplement: Data Sheet 1 — Search terms. [file Data_Sheet_1.docx]

Supplementary Material 1 – Search Terms

# Search Terms Update Literature Search

**Scopus:**

Search string: ( "food" OR "eat" OR "fruit" OR "vegetable" OR "drink" OR "beverage" OR "diet" OR "nutriti*" OR "calorie" ) AND ( "nudg*" OR "choice architect*" OR "behavioral economics" OR "behavioral intervention" ) AND ( "field study" OR "field experiment" ) AND NOT ( "lab study" OR "lab experiment" ) AND ( "selection" OR "consumption" OR "sales" OR "choice" ) AND ( LIMIT-TO ( PUBYEAR , 2019 ) OR LIMIT-TO ( PUBYEAR , 2018 ) )

- 432 results

**PubMed**

Search string: ( "food" OR "eat" OR "fruit" OR "vegetable" OR "drink" OR "beverage" OR "diet" OR "nutriti*" OR "calorie" ) AND ( "nudg*" OR "choice architect*" OR "behavioral economics" OR "behavioral intervention" ) AND ( "field study" OR "field experiment" ) AND NOT ( "lab study" OR "lab experiment" ) AND ( "selection" OR "consumption" OR "sales" OR "choice" )

- 0 additional results

Search strings (according to Cadario & Chandon, 2019): ("food" OR "eat*" OR "fruit" OR "vegetable" OR "drink" OR "beverage" OR "diet" OR "nutriti*" OR "calorie") AND ("nudg*" OR ("choice" AND "architect*"))

- 1 additional result
