# Supplementary material for: Equity Effects of Dietary Nudging Field Experiments: Systematic Review
Source: Front Public Health. 2021 Jul 23;9:668998. doi: 10.3389/fpubh.2021.668998 (PMC8342848; doi:10.3389/fpubh.2021.668998)
Supplement: Data Sheet 2 — Risk of bias (ROBINS-I). [file Data_Sheet_2.docx]

## Supplementary Figure: Risk of Bias Analysis

| **Study** | **Counfounding** | **Selection** | **Intervention Classification** | **Deviations from Protocol** | **Missing Data / Attrition** | **Measurement of Outcomes** | **Reporting Bias** | **Overall Bias** |
| --- | --- | --- | --- | --- | --- | --- | --- | --- |
| Auchincloss et al., 2013 | 2 | 2 | 1 | 1 | 2 | 1 | 2 | 2 |
| Bauer et al., 2021 | 2 | 1 | 1 | 1 | 1 | 1 | 1 | 1 |
| Bollinger et al., 2011 | 2 | 1 | 1 | 1 | 1 | 1 | 2 | 2 |
| Cawley et al., 2020 | 1 | 1 | 1 | 1 | 1 | 1 | 1 | 1 |
| Crockett et al., 2014 | 1 | 1 | 1 | 1 | 1 | 1 | 1 | 1 |
| Elbel et al., 2009 | 1 | 2 | 1 | 1 | 2 | 2 | 3 | 2 |
| Elbel et al., 2013 | 2 | 2 | 2 | 2 | 2 | 2 | 3 | 2 |
| Freedman et al., 2011 | 2 | 2 | 1 | 1 | 2 | 2 | 2 | 2 |
| Krieger et al., 2013 | 2 | 1 | 2 | 1 | 2 | 2 | 2 | 2 |
| Levy et al., 2012 | 3 | 1 | 1 | 2 | 2 | 1 | 1 | 2 |
| Mistura et al., 2019 | 2 | 1 | 1 | 1 | 3 | 1 | 2 | 3 |
| Nagatomo et al., 2019 | 3 | 2 | 1 | 1 | 3 | 1 | 1 | 3 |
| Oliviera et al., 2018 | 1 | 1 | 1 | 2 | 2 | 2 | 1 | 2 |
| Payne et al., 2018 | 3 | 2 | 2 | 2 | 2 | 2 | 1 | 2 |
| Polacsek et al., 2018 | 1 | 1 | 1 | 2 | 1 | 1 | 1 | 2 |
| Salmivaara et al., 2019 | 2 | 1 | 1 | 1 | 2 | 2 | 2 | 2 |
| Thorndike et al., 2014 | 1 | 1 | 1 | 1 | 1 | 1 | 2 | 1 |
| Vanderlee et al., 2014 | 2 | 1 | 1 | 1 | 2 | 2 | 1 | 2 |
| Vermote et al., 2020 | 2 | 1 | 1 | 1 | 1 | 1 | 1 | 1 |

**Supplementary Figure 1.** Risk of Bias Analysis based on Risk of Bias in Non-Randomised Studies of Interventions (ROBINS-I) tool.
